# Toward a Better Paradigm for Head and Neck Cancer Treatment Applying AI (HNC-TACTIC): Protocol for an International Cohort Study of Electronic Health Records

**DOI:** 10.2196/83598

**Published:** 2026-07-13

**Authors:** Hisham Mehanna, Jacobo Rogado, Alejandro Castro Calvo, Víctor González, Francina Aguilar, Dorian Culié, Álvaro Sanabria, Sergio Fabian Zuñiga Pavia, Marta Guix, Sujith Baliga, Roland Giger, Sara-Lynn Hool, Olgun Elicin, Matthaeus Stoehr, Ahmad K Abou-Foul, Melvin L K Chua, Pablo Parente, Andreas Dietz, John R de Almeida, Christian Simon, F Christopher Holsinger, Robert Ferris, Raul Giglio, Kate Hutcheson, David Casadevall, Miren Taberna

**Affiliations:** 1 Institute of Cancer and Genomic Sciences, Birmingham, United Kingdom; 2 Hospital Universitario Infanta Leonor, Madrid, Spain; 3 Hospital Universitario La Paz, Madrid, Spain; 4 Hospital Universitario de Móstoles, Madrid, Spain; 5 Hospital General de Granollers, Barcelona, Spain; 6 Centre Antoine Lacassagne, Institut Universitaire de la Face et du Cou, Nice, France; 7 Department of Surgery, School of Medicine, Universidad de Antioquia-Hospital Alma Mater de Antioquia Medellín, Antioquia, Colombia; 8 Hospital Universitario Nacional de Colombia, Bogotá, Colombia; 9 Hospital del Mar Research Institute, Barcelona, Spain; 10 Massachusetts General Hospital, Boston, MA, United States; 11 Oto-Rhino-Laryngology, Head and Neck Surgery, Inselspital, Bern University Hospital and University of Bern, Bern, Switzerland; 12 Radiation Oncology, Inselspital, Bern University Hospital and University of Bern, Bern, Switzerland; 13 Universitätsklinikum Leipzig, Leipzig, Germany; 14 National Cancer Centre Singapore, Duke-NUS Medical School, Singapore, Singapore; 15 Hospital Universitario de A Coruña, A Coruña, Spain; 16 University of Toronto, Toronto, ON, Canada; 17 Centre Hospitalier Universitaire Vaudois (CHUV), Lausanne, Switzerland; 18 Stanford University School of Medicine, Stanford, CA, United States; 19 UPMC (University of Pittsburgh Medical Center) Hillman Cancer, Pittsburgh, PA, United States; 20 Hospital Roffo, Buenos Aires, Argentina; 21 MD Anderson Cancer Centre, Houston, TX, United States; 22 Savana Research, S.L, Calle Larra, 12 - BJ IZ, Madrid, 28004, Spain, 34 910696902

**Keywords:** HNSCC, predictive factors, overall survival, immunotherapy, electronic health records, natural language processing, artificial intelligence, head and neck squamous cell carcinoma

## Abstract

**Background:**

Head and neck squamous cell carcinomas (HNSCCs) cause considerable morbidity and mortality. Multimodal treatment strategies can cause significant toxicity, and therapy options are limited for recurrent disease. Immunotherapy has emerged as a promising approach. However, patient response variability underscores the need for better predictive markers.

**Objective:**

This study aims to use artificial intelligence to develop two predictive models in patients with HNSCC to assess (1) progression or recurrence following primary curative treatment and (2) long-term survival after immunotherapy schemes in recurrent and metastatic disease. This study will also describe the characteristics of patients with early, locally advanced, and recurrent or metastatic cancers.

**Methods:**

This is a retrospective, observational study of data captured in electronic health records (EHRs) from participating hospitals between January 1, 2014, and December 31, 2021. This study’s population comprises adults diagnosed with HNSCC at any stage. Study variables, including demographics, comorbidities, clinical variables, treatments, and outcomes, will be extracted using EHRead, a technology that applies natural language processing and machine learning to extract and analyze structured and unstructured clinical information in deidentified EHRs. Predictive models based on dynamic risk stratification for treatment response and progression or recurrence will be developed using multivariable logistic regressions, decision tree classifiers, and random forest approaches. Descriptive and outcome analyses will be shown for different anatomic subsites and stratified by stage and treatment.

**Results:**

This study began enrolling sites in July 2021 and is currently ongoing. By December 2025, data from 10 centers has been collected, comprising a total of 151,934,990 EHRs from 2,159,719 patients.

**Conclusions:**

Development of predictive models using artificial intelligence will advance clinical understanding of HNSCC to improve patient outcomes.

## Introduction

### Background and Rationale

Head and neck cancer (HNC) ranks as the seventh most prevalent malignancy worldwide, comprising around 4.5% of all cancer cases and causing over 600,000 deaths each year, according to Global Cancer Observatory estimates [1]. Most of these cases are classified as head and neck squamous cell carcinoma (HNSCC) [2].

For patients with early-stage human papillomavirus (HPV)–unrelated HNCs, surgery or radiotherapy alone is typically recommended. However, for HNSCC, multimodality treatment combining two or more approaches, such as surgery, radiotherapy, chemotherapy, and immunotherapy, is generally advised [3]. Early-stage patients have a favorable prognosis, with a median 5-year overall survival (OS) rate of 70% [4]. In cases of locally advanced disease, functional sequelae after surgery and radiotherapy can be significant, and despite multimodality therapy, up to 60% of patients experience locoregional recurrence and/or metastasis within 3 years [5-8]. In patients with metastatic disease, there is significant variability, with reported figures reaching up to 4% OS at 5 years in cases of patients with multiple metastases [9,10]. Improved survival in developed countries is influenced by several factors, including the increasing prevalence of HPV-related cases with better outcomes, advancements in surgical and radiotherapy techniques, and the introduction of immunotherapies such as checkpoint inhibitors [11].

Most patients with recurrent or metastatic HNSCC disease are ineligible for salvage therapy with radical intent, and palliative therapy is the only treatment option [8]. The introduction of immunotherapy in patients with recurrent or metastatic disease who progress during or after platinum-based chemotherapy has resulted in longer survival [12]. Moreover, recent studies evaluating the efficacy of immunotherapy as a first-line treatment in recurrent or metastatic patients with HNSCC have demonstrated that immunotherapy not only increases the median OS from 11 to 14.7 months in some settings, but has also led to long-lasting survival, with 20%‐30% of patients living beyond 3 years [5,7,13]. While some factors, such as the expression of PD-L1 (programmed death-ligand 1) or the degree of tumor lymphocyte infiltration, could play an important role in treatment outcomes, the differential response to immunotherapy is not completely understood [7].

Overall, low survival rates in combination with significant late toxicities caused by current multimodal treatment strategies underscore the urgent need to define better predictors of treatment response in patients with HNSCC [14]. To achieve this, it is essential to better characterize the different stages of the disease and identify the best approaches in terms of treatment outcomes, organ function preservation, and quality of life. In recent years, the application of artificial intelligence (AI) tools, specifically clinical natural language processing (cNLP) and machine learning (ML), has shown great promise for addressing these challenges. Both cNLP and ML techniques offer several benefits in the field of health care, including minimizing selection bias and missing data, the ability to incorporate a wide range of variables, and the requirement of fewer human resources compared to manual extraction [15]. By leveraging large datasets and advanced algorithms, these technologies can analyze and extract valuable insights from free text in medical records. Additionally, in the field of AI, predictive models are poised to revolutionize clinical decision-making in oncology, paving the way for a more personalized approach to treatment. These models, with their remarkable ability to identify treatment responders, are the key to minimizing side effects and maximizing treatment success. In the context of HNSCC, the development of predictive models for treatment outcomes offers significant promise, providing crucial insights into disease prognosis and offering valuable guidance for clinical decisions, ultimately enhancing the care of patients with HNSCC [16].

### Objectives

The HNC-TACTIC study (Toward a Better Paradigm for Head and Neck Cancer Treatment Applying Artificial Intelligence) aims to describe the clinical characteristics and real-world outcomes of patients with HNSCC at all disease stages, and to develop 2 predictive models using AI techniques such as cNLP and ML. Specifically, the primary objectives of the HNC-TACTIC study are to develop 2 predictive models based on dynamic risk stratification to (1) assess the risk of disease progression or recurrence following a primary curative treatment of early and locally advanced HNSCC and (2) identify patients’ features that predict long-term survival after immunotherapy in recurrent and metastatic patients with HNSCC.

Secondary objectives will focus on a descriptive analysis of the real-world outcomes of patients with HNSCC at all disease stages. Eventually, the exploratory objectives of this study will characterize the nonfrequent HNC population, encompassing patients with tumors in rare anatomical sites within the head and neck region, such as the nasopharynx, nasal cavity, paranasal sinuses, and salivary glands, as well as those with histological subtypes other than squamous cell carcinoma. All study objectives are represented in Textbox 1.

Textbox 1. **Detailed description of study objectives.**

Primary objectives

- PO1 (primary objective): to develop a predictive model for the risk of recurrence or disease progression following a primary curative treatment in patients with head and neck squamous cell carcinoma (HNSCC) with early and locally advanced disease.
- PO2: to develop a predictive model for long-term survival in recurrent and metastatic patients with HNSCC treated with immunotherapy in second line.

Secondary objectives

- In all patients with HNSCC
  - SO1 (secondary objective): to describe overall survival (OS) in patients with different tumor stages and treatment approaches by primary tumor location.
- In early or locally advanced patients with HNSCC
  - SO2: to describe demographics, clinical characteristics, and treatments.
  - SO3: to describe follow-up patterns: health care resource utilization (HCRU), and recurrence.
  - SO4: to evaluate the impact of treatment approaches, including adverse events and HCRU (only in locally advanced patients with HNSCC).
  - SO5: to compare OS in patients with different treatment approaches and different types of conservative treatment by human papillomavirus status (only in locally advanced oropharyngeal cancers).
  - SO6: to compare the demographic and clinical characteristics of different types of responders by human papillomavirus status (only in locally advanced oropharyngeal cancers).
- In patients with HNSCC with recurrent or metastatic disease
  - SO7: to describe demographics, clinical characteristics, and treatments.
  - SO8: to describe demographics, clinical, and tumor characteristics of long-term survivors by treatment approach.
  - SO9: to describe the impact of immunotherapy treatment and use, including OS and HCRU.

Exploratory objectives

- EO1 (exploratory objective): to describe the demographics, clinical characteristics, and treatment of patients with nonfrequent head and neck cancer.

## Methods

### Study Design

This protocol describes a multicenter, international, retrospective study that will analyze clinical information captured in the electronic health records (EHRs) of all patients with HNC from each participating site. This study is currently enrolling centers, anticipating 11 participating sites from 8 different countries (Spain, France, the United Kingdom, Germany, Switzerland, the United States, Canada, and Colombia). Structured and unstructured data will be captured from the EHRs of patients who attended the participating hospitals during this study’s period, spanning January 1, 2014, through December 31, 2021.

**Figure 1.**
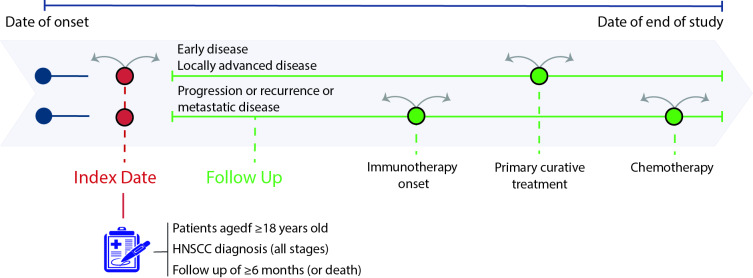
Time windows for data analysis. For all patients, the index date (red dot) is defined as the time point within this study’s period when they fulfill all inclusion criteria and no exclusion criteria. The follow-up (green line) comprises the period between the index date and the last EHR available within this study's period. The time for analysis of selected endpoints for the predictive models is shown for patients in the different study subgroups (green dots). EHR: electronic health record; HNSCC: head and neck squamous cell carcinoma.

This study will include all adult patients diagnosed with any stage of HNC, including not only HNSCC but also nonfrequent tumors (nasopharynx, nasal cavity, paranasal sinus, or salivary gland) in any of the participating hospitals. The time of inclusion for each patient, referred to as the index date, is defined as the time point within this study’s period when all inclusion criteria and no exclusion criteria are met. Patients who meet these criteria outside this study’s period will be excluded from further analyses. This study’s variables will be analyzed at baseline (variable windows around the index date) and during a follow-up, between the index date and the last EHR available within this study’s period (Figure 1). Accordingly, 2 different types of analysis will be performed: cross-sectional analyses at inclusion time and longitudinal analyses to include follow-up data.

### Study Population

The screening set will include all EHRs from patients who attended any of the participating hospitals during this study's period. Among them, patients with a confirmed diagnosis of HNC will comprise the source population. Patients from the source population who meet all the following inclusion criteria and none of the exclusion criteria will be included in the final study population:

The inclusion criteria were being aged ≥18 years and having HNSCC or nasopharynx, nasal cavity, paranasal sinus, or salivary gland tumor diagnosis.

The exclusion criteria were patients with a follow-up period shorter than 6 months (except for those patients who progress or die during this time).

The EHRead technology developed by Medsavana will screen all patients’ EHRs within the source population, and those that qualify for this study will be selected through filters based on the inclusion and exclusion criteria listed before. All these filters will be reviewed by medical research experts with broad cNLP experience to ensure that only the data from patients who qualify for this study are analyzed.

### Study Subpopulations and Stratifications

**Figure 2.**
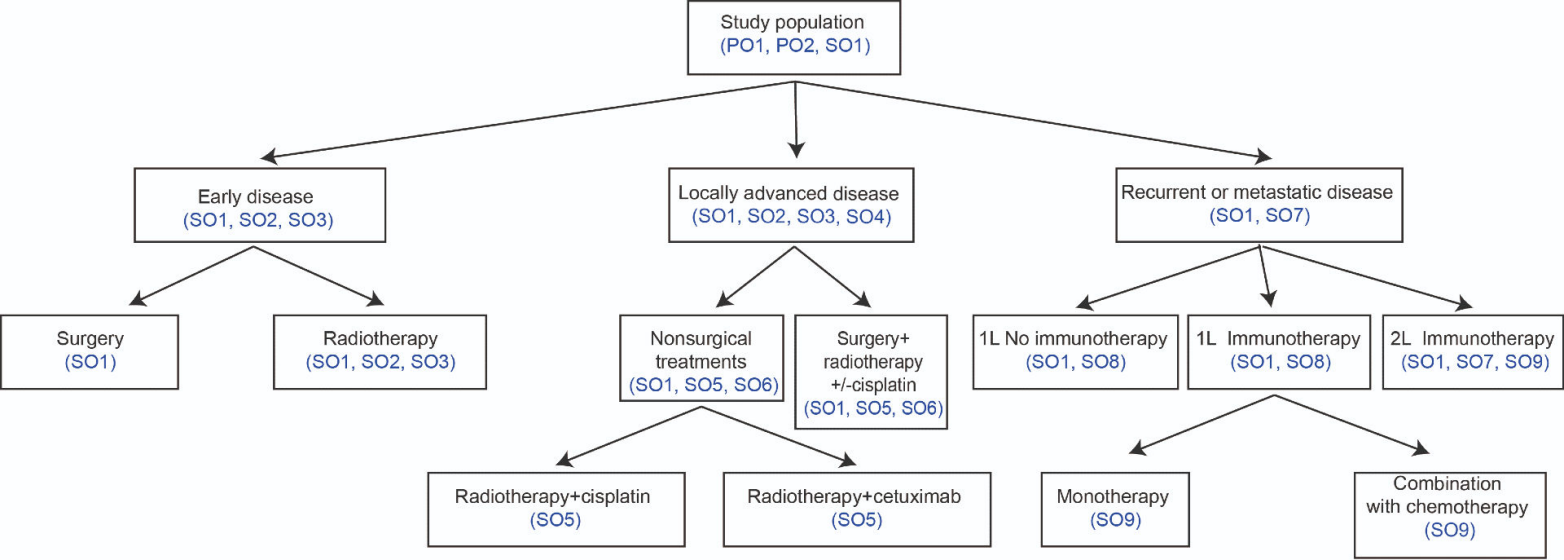
Population flowchart. Schematic representation of this study's population and subpopulations indicating stratification according to disease stage and treatment for the primary and secondary objectives. 1L: first line of treatment; 2L: second line of treatment; EO: exploratory objective; PO: primary objective; SO: secondary objective.

Within the overall study population, we will differentiate between patients with HNSCC and patients with other nonfrequent HNC, such as nasopharynx, nasal cavity, paranasal sinus, and salivary gland tumors. Subpopulations and analysis strata based on disease stage or extension and/or treatment approach will be defined to answer this study's objectives. Study populations, subpopulations, and stratifications are shown in Table 1 and represented in the flow chart in Figure 2.

**Table 1.** Description of analysis populations, subpopulations, and stratifications. Study objectives for each stratification approach are indicated in parentheses.

Study population	Study subpopulation	Stratifications
Patients with HNSCC^a^	Early and locally advanced HNSCC	Primary tumor location (oral cavity, oropharynx, larynx, and hypopharynx; SO1^b^). Treatment approach (conservative treatment or surgery; SO1, SO4, SO5). Type of nonsurgical treatment (cetuximab+radiotherapy or platinum-based chemotherapy+radiotherapy; SO5). HPV^c^ status for oropharynx patients (positive, negative, and unknown; SO5, SO6). Type of responders (exceptional or poor; SO6).
Patients with HNSCC	Recurrent or metastatic HNSCC	Primary tumor location (oral cavity, oropharynx, larynx, and hypopharynx; SO1). Treatment approach (immunotherapy or nonimmunotherapy; SO8, SO9). Immunotherapy use (monotherapy or combination with chemotherapy; SO9).
Nonfrequent head and neck tumors	Not applicable	Primary tumor location (nasopharynx, nasal cavity, paranasal sinus, and salivary gland; EO1^d^).

a HNSCC: head and neck squamous cell carcinoma.

b SO: secondary objective.

c HPV: human papillomavirus.

d EO: exploratory objective.

### Data Source and Extraction of Study Variables

This will be a data-driven study concerning the extraction and analysis of this study's variables registered in the EHRs by the physicians during routine clinical practice. The data source will be unstructured free-text information (and structured if available) in patients’ EHRs, including outpatient clinic reports, discharge reports, emergency reports, prescriptions, laboratory results, and other medical reports. No research-related documents will be collected for this study.

Patient characteristics will include age, sex, and toxic habits such as tobacco and alcohol use. Clinical characteristics will include comorbidities, baseline clinical status, symptoms and signs, tumor location, stage, HPV status, treatment, and treatment response. Follow-up characteristics will include healthcare resource utilization, recurrence or progression, and survival. Outcome definitions have been prespecified and will be applied consistently across all analyses. For PO1, recurrence or progression is defined as the first documented evidence of locoregional recurrence, distant metastasis, or disease progression occurring after completion of primary curative treatment. The time 0 for all recurrence or progression analyses will be the end date of primary curative treatment. For PO2, long-term survival will be defined as survival ≥18 months after initiation of immunotherapy in patients with recurrent or metastatic HNSCC. The time 0 for survival analyses in this cohort will be the date of immunotherapy initiation. Missing data are expected due to the retrospective nature of this study. Table 2 shows the filters to be applied to define the most important described clinical entities. All variables to be analyzed in this study are detailed in Tables S1-S3, listed by objective and classified by patient population, category, and time point for analysis.

**Table 2.** Definition of study variables.

Clinical entities	Definition
Tumor location	Oral cavity, oropharynx, larynx, and hypopharynx (HNSCC^a^). Nasopharynx, nasal cavity, paranasal sinus, and salivary gland (nonfrequent HNC^b^).
Tumor stage	For oral cavity, larynx, hypopharynx, and p16 (HPV^c^)-negative oropharynx cancer Early stage: I, II Locally advanced: III, IVA, IVB Metastatic: IVC For p16 (HPV)-positive oropharynx cancer stages Early stage: I, II Locally advanced: III Metastatic: IV
HPV status	Positive, negative, or unknown result according to HPV DNA testing or p16 expression by immunohistochemistry
Treatment	Early stage: surgery, radiotherapy, concomitant chemoradiotherapy Locally advanced: surgery, radiotherapy, concomitant chemoradiotherapy, neoadjuvant chemotherapy Recurrent or metastatic disease: chemotherapy, chemoimmunotherapy, or immunotherapy alone
Response	Exceptional responder: patient receiving curative treatment, with no documented recurrence or progression during follow-up. Poor responder: patient receiving curative treatment, with documented recurrence or progression within 18 months after completion of treatment.
HCRU^d^	Departments in charge, number of outpatient visits and hospitalizations within each department, images, and histopathologic tests.
Progression or recurrence	Progression or recurrence: first documented evidence of locoregional recurrence detected by physical examination, distant metastasis, or disease progression occurring after completion of primary curative treatment, identified through EHR^e^ free text. Detection of the terms “recurrence,” “progression,” “metastasis,” or any of their alternative names in free text on clinical notes or radiology reports, as well as “physical examination” or any of its alternative names in patients without imaging testing.
Survival	Overall survival: time from diagnosis of HNC to death from any cause or last available follow-up. Long-term survival (in patients treated with immunotherapy): Survival beyond 18 months from initiation of first-line immunotherapy. Long-term survivors are defined as patients alive at the 18-month landmark.

a HNSCC: head and neck squamous cell carcinoma.

b HNC: head and neck cancer.

c HPV: human papillomavirus.

d HCRU: health care resources utilization.

e EHR: electronic health record.

### cNLP Pipeline

This study will be conducted following the guidelines outlined in this study's protocol and statistical analysis plan, ensuring a prespecified and systematic approach. This framework will guide data extraction from each hospital, the development of cNLP processing, and the execution of analyses to meet this study's objectives.

EHRead is a powerful cNLP pipeline capable of processing multilingual free text, analyzing, and meaningfully interpreting clinical records regardless of the EHR system. It captures and extracts clinically relevant information from EHR free-text, providing information in a structured database [17-23]. The extraction of clinical entities, including those needed to define population, interventions, and outcomes, follows a supervised human-in-the-loop approach. First, clinical experts define the entities, relationships, and terminology required to construct each variable. In this context, all study variables are predefined and mapped to clinical terms using standardized classifications, including the Systematized Nomenclature of Medicine Clinical Terms scaffold [24,25], the anatomical therapeutic chemical classification, and the LOINC (Logical Observation Identifiers Names and Codes) [25]. EHRead will then use cNLP models, such as named entity recognition and named entity linking (NER-NEL), to capture and extract those clinical terms relevant to this study's disease, linking them to the corresponding clinical concepts. NER-NEL models are trained on comprehensive sets of terms, alternative names, and acronyms, and refined iteratively through manual annotation of documents from all participating hospitals to identify false positives, false negatives, and center-specific terminology. Detected entities are then contextualized, identifying term modifiers using models for negation, temporality, attributes, and adjectives, among others. The same algorithms are applied uniformly across all hospitals using EHRead technology, ensuring standardized data extraction and minimizing data heterogeneity. The accuracy of clinical terms and study variables will be reviewed and validated by 2 oncologists.

### cNLP Execution and Performance Evaluation

#### Overview

After the terms related to the HNSCC panel are approved by expert oncologists, the performance of NER-NEL in capturing eligible terms for cNLP assessment will be evaluated. This evaluation will focus on terms identified and extracted directly through cNLP detection. However, certain clinical terms relevant to this project will be excluded from the cNLP performance assessment. These terms will either be obtained from structured data (eg, age, sex, and specific drugs), analyzed based on the detection of their independent components (eg, drug combinations), or require specific integrity checks to ensure that all variables are extracted according to the expected metrics. Finally, all databases from each participating hospital will be merged into a single aggregated database. The resulting database will include clinical terms detected by EHRead technology for predefined clinical variables. A specialized team will convert the dataset into an analysis-ready format, and standard data quality checks will be applied to assess missing data and heterogeneity.

To assess term-level precision, recall, and *F*_1_-score, 2 separate annotation projects will be conducted [13]. The first project will focus on a targeted selection of sentences for each alternative name, abbreviation, and acronym within the variable panel. The second project will involve selecting full documents using a self-led infection control assessment tool, based on a comprehensive set of key or critical terms [10]. The self-led infection control assessment tool will ensure that the performance metrics for cNLP system evaluations are both robust and representative by relying on a statistically validated gold standard.

External validation will guarantee that the quality of variable extraction and the generalization capability of the EHRead technology objectively meet expectations [23]. This validation will be performed for a selected set of key variables of this study (variables defining this study's population and primary objectives) and carried out by external annotators (local coinvestigators in each participating hospital) following a peer-reviewed Savana method [26]. It will measure the interannotator agreement and ensure the consistency of the guidelines and the reliability of the annotated parameters.

Performance metrics will be calculated as follows:

Precision (*P*) = *tp*/(*tp* + *fp*); indicates the accuracy of the information the system retrieves.

Recall (R) = *tp*/(*tp* + *fn*); indicates the amount of information the system retrieves.

*F*_1_-score = (2 ×  P × *R*)/(*P* + *R*); overall performance indicator of information retrieval.

**Figure 3.**
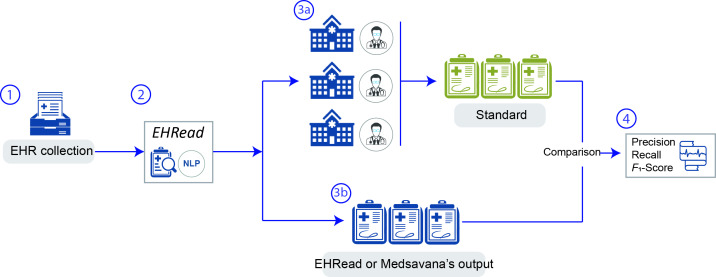
Execution and evaluation of EHRead. A set of EHRs (1) collected via EHRead (2) is annotated by physicians to generate a standard (3a) and compare it to this study's results (3b), obtaining precision, recall, and *F*_1_-scores to evaluate the quality of EHRead output (4). EHR: electronic health record.

In all cases, *tp* is the number of true positives, *fn* is the number of false negatives (ie, records incorrectly not retrieved), *fp* is the number of false positives (ie, records incorrectly retrieved), *P* is precision, and R is recall. A precision and *F*_1_-score value of 0.8 or higher will be considered acceptable for key or critical terms. If lower metric values are obtained, the annotation project will be re-executed until precision reaches the acceptable threshold. The external validation will lead to the creation of a standard to which the EHRead technology’s (cNLP system) variable detection will be compared (Figure 3).

#### Study Population Validation

The evaluation of the validity and specificity of definitions for the population, intervention, and outcomes involves 3 main steps: generating an independent sample, establishing a reference standard, and comparing definitions against this standard. First, the independent sample will be selected using structured data to enhance the presence of the target population. Sample size calculations will follow established methodologies for categorical [27] and continuous [28] variables. A clinical adjudication committee of 3 independent medical experts will serve as the reference standard. The committee members will not have access to the knowledge of the rules governing the natural language processing algorithms. Further, 2 doctors will independently assess cases, with a third acting as a coordinator-reviewer in case of disagreement. Persistent discrepancies will be resolved through a consensus meeting, following Kradjian et al [29]. Additionally, the coordinator-reviewer will perform interim analyses to assess intra- and interreviewer agreement. Finally, the generated definitions (natural language processing algorithms + clinical data science rules) will be evaluated using classification statistics, including the area under the receiver operating characteristic curve, sensitivity, specificity, positive predicted value, and negative predicted value, to measure their accuracy in identifying cases compared to the clinical reference standard.

### Patient and Public Involvement

This study is a retrospective analysis using an anonymized database resulting from the information extracted from EHRs. As such, patients and members of the public were not involved in this study's design and will not participate in its conduct, data reporting, or dissemination plans. The reuse of information from routinely collected clinical data did not require direct patient input, and no identifiable personal information was accessed or used during the analysis phase of this study.

### Ethical Considerations

#### Overview

This protocol followed SPIRIT (Standard Protocol Items: Recommendations for Interventional Trials) recommendations for clinical trial protocol formulation.

#### Ethics Approval

All participating centers are required to submit this study's protocol to their respective Research Ethics Committee and obtain approval before initiating any study-related activities. As of December 2025, a total of 10 centers across Colombia, France, Germany, Spain, and Switzerland have completed this process and received ethical approval by the Research Ethics Committee from A Coruña-Ferrol (2021/426) and the Germany Ethik-Komission an der Medizinischen Fakultät der Universität Leipzig (253/22-ek).

#### Informed Consent Statement

In this study, there will not be a data reporting form. The applied methodology involves a secondary use of the data contained in the EHR’s through the combination of existing structured and unstructured data; therefore, it does not involve direct interaction with this study's participants. No patient’s informed consent will be required in this study. The exemption of such consent will be requested by the ethics committees, as applicable. Patients will not receive any compensation for their participation in this study.

#### Patient Information and Data Anonymization

##### Overview

To ensure confidentiality, data will be anonymized before being included in the research process. By carrying out this study with an anonymized database, there would be no processing of personal data and, therefore, no need to comply with the requirements included in the data protection regulations, among them, the lawfulness of the data processing as well as the information to be provided to the data participants.

##### Data Anonymization Process

This study will be conducted using a fully anonymized database. To ensure compliance with data protection standards, a multistep anonymization strategy is implemented. First, each participating site will perform a pseudonymization process on its own EHRs before data transfer. This includes the removal of personal identifiers (eg, names, patient IDs, and birth dates), which are replaced by randomly generated codes. The mapping table linking original and pseudonymized identifiers will be securely stored at each site and will not be accessible to Medsavana (the technological service provider responsible for the extraction, structuring, validation, and enforced pseudonymization of EHR data from each participating site) at any point.

Once the pseudonymized data is received, Medsavana will apply cNLP techniques to extract clinically relevant variables defined in this study's protocol. These variables will be structured using standardized clinical ontologies, such as Systematized Nomenclature of Medicine Clinical Terms, and the resulting dataset will contain no free text. During this process, further anonymization measures will be applied to any remaining identifiers. For example, birth dates will be standardized by replacing the actual day with the 15th of the month, and a 1-way salted hash function will be applied to the document, episode, hospital, and patient identifiers. This process ensures that the data are irreversibly anonymized and no longer traceable to individual patients.

The anonymized datasets from each site will then be aggregated into a single, study-wide database, which will be the only version shared with Savana Research, the company responsible for conducting the clinical analyses according to the protocol and statistical analysis plan. At no point will Savana Research have access to raw or pseudonymized data. Once data integration is complete and the final database is verified, Medsavana will disable access to any intermediate data, guaranteeing the irreversibility of the anonymization process.

### Institutional Review Board Statement

This study will be conducted in accordance with legal and regulatory requirements, as well as with scientific purpose, value, and rigor, and following generally accepted research practices described in the International Society for Pharmacoepidemiology Guidelines for Good Pharmacoepidemiology Practices and applicable regulatory requirements, and the International Council for Harmonization of Technical Requirements for Pharmaceuticals for Human Use Guideline for Good Clinical Practice, the Declaration of Helsinki in its latest edition, and applicable local regulations. There will be prospective approval of this study's protocol, protocol amendments, and other relevant documents from the relevant institutional review boards and the independent ethics committee. All amendments to the protocol will be subject to appropriate review and approval, in accordance with local regulations. At the end of this study, when required by local regulations, the Independent Ethics Committee will be notified of study completion.

### Dissemination Policy

In line with Good Publication Practices and ICMJE (International Committee of Medical Journal Editors) guidelines, the database owner retains the right to publish multicenter data without needing approval from participating physicians. Participant physicians agree not to publish site-specific data until the combined study results are submitted for publication. Within 12 months for final data availability. Authorship will follow the ICMJE standards, requiring substantial contributions to study design, data analysis, or interpretation, critical paper review, and final approval.

### Statistical Analysis

#### Sample Size

For the analysis of immunotherapy’s long-term effects on recurrent HNSCC survival (SO9), a minimum sample size of 808 is estimated necessary to analyze patient survival, considering findings reported in the Checkmate 141 trial [6]. This approach considers a 40% recurrence prevalence, a 4:1 ratio between standard chemotherapy and immunotherapy groups, a 2-year survival of 0.02 in the reference group, and a minimum expected relative risk of 0.60 for the intervention group. In addition, a dropout rate of 1%, a risk *α* of .05, and a risk *β* of .2 were accepted in a 2-sided test. However, unlike the Checkmate 141 trial, TACTIC’s retrospective and observational design prompts consideration of additional statistical adjustments and matching procedures to reflect conditions encountered in routine clinical practice. Based on the most current literature on comparative effectiveness analysis with observational data, it seems reasonable to anticipate an oversampling of 4 to 8 times over the calculated number to address potential sample attrition postmatching procedures [30]. Accordingly, this would correspond to an estimated range of 3200‐6400 patients for comparative effectiveness analyses of survival outcomes.

Primary objective 2 is more ambitious because it aims to develop a predictive model for the immunotherapy-treated recurrent or metastatic patient with HNSCC subgroup, which involves additional theoretical assumptions about expected model performance, predictor parameters, and event prevalence [31]. Given that we were unable to identify any previously published model in this specific clinical setting and assuming (1) a model performance based on area under the curve of 0.65 (acceptable), 0.75 (good), or 0.85 (excellent); (2) an initial number of (training) predictor parameters of 20, 30, and 40, respectively; and (3) a long-term survival rate of 20% in patients treated with immunotherapy, the minimum sample sizes to develop a predictive model would look as shown in . Based on these assumptions, the estimated sample sizes reported in Table S4 (Multimedia Appendix 1) represent theoretical minimum requirements for optimal model development.

These estimates should be interpreted as theoretical benchmarks rather than fixed recruitment targets, as the final sample size will be determined by data availability in the participating centers. The final number of patients available for predictive modeling will depend on the proportion of patients with recurrent or metastatic disease and immunotherapy exposure across participating centers during this study's period. In this context, model complexity, number of predictors, and modeling strategies will therefore be adapted to the available sample size to ensure methodological robustness and minimize overfitting. Moreover, if required to achieve sufficient statistical power or adequately populate specific subgroups, additional centers may be incorporated and/or this study's period may be extended, in line with feasibility assessments and data availability across sites.

#### Statistical Analysis Framework

This study’s protocol includes both cross-sectional and longitudinal objectives that require descriptive analyses and predictive modeling. Summary statistics of all study variables will be calculated for all included populations and strata (mainly tumor stage, location, treatment approach, HPV status, and treatment response). All statistical analyses will be performed using the latest versions of R software (R Foundation) or Python (Python Software Foundation).

#### Descriptive Analyses

Frequency tables will be produced for Boolean and categorical variables, whereas continuous variables will be summarized by extracting the mean, SD, median, minimum, maximum, and quartiles of each variable. Further, 2-sided 95% CIs will be provided for stratified descriptive analyses. For categorical variables, CI will be calculated as the estimated point ±the product of a critical value (z), derived from the standard normal curve, and the standard error of the estimated point. For continuous variables, CI will be calculated as the mean ±1.96 times the SD for normally distributed variables and as the median ±2.5 and 97.5 percentiles for nonnormally distributed variables. Time to event analyses will be performed by calculating incidence rates (frequency of an event per person-time of observation) and by the Kaplan-Meier method. Transformations will be considered when appropriate.

For descriptive analyses, missing data will be handled according to the nature of the data collection process (see description of Medsavana’s EHRead technology in the section cNLP Pipeline). Missing data imputation will occur only for certain types of Boolean variables, such as comorbidities or symptoms, which are assumed to be reported if presented by the patient. For these variables, their absence in patients’ EHRs will be imputed as a true absence (ie, the patient lacks that comorbidity or symptom). For multilevel (≥3) categorical variables and certain variables such as lifestyle factors (eg, smoking or drinking habits), their absence will not be imputed, and the number of patients with missing data will be reported. For numeric variables, no missing data imputation strategies are planned in the descriptive analysis, but will be considered for predictive modeling.

#### Predictive Models

Before generating the predictive model, the database will be prepared based on the findings derived from the secondary objectives. Once the maximum number of predictors to train the models has been calculated (section Data Source and Extraction of Study Variables), train or test datasets will be constructed to estimate the models and to evaluate their performance and stability. Data splitting techniques may include bootstrap resampling and/or k-fold cross-validation. In addition, several measures may be undertaken to ensure that the sample size is adequate for model training [31], minimizing model overfitting:

- The distribution of events across time will be analyzed to determine which discrete time points are more appropriate to maximize the number of candidate predictors for classification-based algorithms.
- The proportion of events and censored (loss of follow-up) patients during the follow-up period will be analyzed to estimate the sample size needed for survival-based algorithms.

Primary objective 1 aims to determine the risk of recurrence or disease progression in the early stages and in locally advanced disease patients with HNSCC. Dynamic risk stratification will be approached by training a different model at each key time point within a patient’s disease trajectory. Key time points will be (1) at diagnosis, (2) three months after a primary curative treatment is finished, and (3) one year after primary curative treatment is finished. The total number of models may be adjusted depending on data availability and granularity. Primary objective 1 outcomes will be assessed both as binary variables (recurrence or progression rates at discrete time points) and as time-to-event. Thus, different model families (classification-based and survival-based) will be trained based on outcome definitions.

Primary objective 2 aims to determine the most important factors associated with a positive response to immunotherapy in patients with recurrent and metastatic HNSCC. First-line immunotherapy patients will be treated as a separate cohort or as a covariate for the model, depending on the final sample size for each setting. Outcomes will be assessed both as binary variables (recurrence, progression, or death rates at discrete time points) and as time-to-event. Candidate predictors will include baseline variables and the selection of postbaseline variables, such as treatment, early toxicities, and response. The decision to select and include postbaseline variables as predictors will be based on secondary objective 2 findings.

To select the appropriate imputation method to handle missing data in important predictors, a careful evaluation of the missing data patterns will be performed, including the availability of auxiliary variables and the feasibility of correct specification of the imputation model [32]. Briefly, missing data evaluations will include (1) description of the amount of missing information for each variable, (2) description of the missing value combination patterns across variables, and (3) investigation of whether the probability of being a complete case depends on the outcome and/or other characteristics. For multiple imputation, an imputation method and a set of predictors will be selected for each variable. The number of imputations required will be determined using the fraction of missing information [33].

Model performance will be measured in terms of the area under the curve of the receiver operating characteristic, and through different metrics derived from the confusion matrix (eg, precision, recall, or *F*_1_-score). For linear models, internal validation will be performed by bootstrap [34] to estimate the optimism (level of model overfitting) to correct the measures of the predictive apparent performance. For nonlinear (ML-based) models, k-fold cross-validation will be used for model training and evaluation.

#### Bias Assessment and Mitigation Strategies

To address potential sources of bias inherent to the use of multicenter, multilingual EHRs, several complementary strategies will be implemented. First, to minimize center-level variability in documentation quality or EHR structure, all participating sites undergo a feasibility assessment before data extraction, followed by language-specific cNLP training and center-specific cNLP validation to ensure consistent identification of clinical concepts across institutions. Second, descriptive cross-center comparisons will be conducted to evaluate heterogeneity in patient demographics, clinical characteristics, follow-up patterns, and outcome distributions. These analyses will support the detection of center-specific differences that could influence comparative or predictive analyses. Finally, predictive models will incorporate internal validation procedures that explicitly assess generalizability across institutions, including leave-one-center-out cross-validation, enabling the evaluation of model performance when entire centers are systematically excluded from training. Together, these measures aim to reduce information bias, mitigate center-level heterogeneity, and enhance the robustness, interpretability, and transportability of study findings across diverse real-world clinical settings.

## Results

This study began enrolling participating sites in July 2021 and remains ongoing. As of December 2025, data from 10 centers have been collected, comprehending a total of 151,934,990 EHRs from 2,159,719 patients. Enrollment of additional centers is continuing, and further data accrual is anticipated as this study progresses. Initial data exploration and quality checks are currently underway to assess completeness, consistency, and feasibility for planned analyses. These preliminary analyses have been reported in 2 conference communications to ESMO in 2024 and 2025 [35,36]. A first manuscript with this study’s first results is planned by the end of 2026.

## Discussion

### Principal Findings

HNSCC is a significant global health issue with a global incidence that has been increasing, particularly in younger populations, with a predicted 30% annual increase in incidence by 2030 [37]. This tendency is partially due to changes in lifestyle factors, such as increased alcohol consumption and tobacco use.

HNSCC presents 5-year survival rates that vary by disease stage at diagnosis, ranging from 86.6% for patients with early-stage, 69.1% for locally advanced, and 39.3% for metastatic disease [10]. Despite advances in numerous therapeutic modalities, these rates have not significantly improved, and current medical and research efforts focus on immunotherapy approaches, targeting immunological checkpoints as well as other signaling pathways [38].

This protocol aims to describe the clinical characteristics and outcomes of patients at all HNSCC stages by analyzing routinely collected data from the EHRs. Indirectly, this protocol also seeks to demonstrate the feasibility of automated clinical data extraction using cNLP and ML techniques in the context of HNC. By applying these methods to EHRs, this study aims to show that clinically relevant information can be systematically and reproducibly identified, structured, and analyzed, supporting the use of advanced computational approaches in oncological clinical research and real-world evidence studies. This study will focus on developing 2 predictive models to support clinical interpretation and discussion, one for selecting patients with a high risk of progression or recurrence after radical treatment, and a second one for the identification of patients with long-term survival outcomes following immunotherapy in recurrent or metastatic HNSCC. Both models can provide valuable insights for clinical decision-making and wield a significant impact on patient management. These predictive models are intended for prognostic stratification and risk estimation, providing clinically interpretable outputs that may support multidisciplinary decision-making. They are not designed to function as autonomous clinical decision support systems, and their use is complementary to clinical judgment and existing treatment guidelines.

ML has been highly effective in predicting various types of cancer, demonstrating its potential in reducing cancer mortality rates [39-41]. AI has emerged as a promising option to improve health care accuracy and patient outcomes, and its current applications in oncology include risk assessment, early diagnosis, patient prognosis estimation, and treatment selection based on deep knowledge [42]. These significant advances in the oncology field because of ML have raised the overall interest of the medical community in AI. In this regard, cNLP and ML emerge as encouraging tools for real-world data extraction, complementing AI’s potential. This is mainly due to their capability to extract more comprehensive and informative data, as well as to their ability to make inferences to identify the events or variables of interest.

### Strengths and Limitations of This Study

#### Strengths

##### Innovative Use of AI

This is the first multilingual, multiregional real-world data study of patients with HNSCC that applies AI methods, including ML, cNLP, and multivariate modeling to real-world clinical data, with a focus on the automated extraction of clinically meaningful variables. This deep extraction of variables may enable better characterization of patients with HNSCC.

##### Comprehensive Data Source

Unlike structured data such as *ICD* (*International Classification of Diseases*) codes, which offer a limited and often superficial view of the clinical context, the use of free-text clinical narratives enables a much deeper and more nuanced extraction of patient information.

##### Descriptive Characterization

Patients with HNSCC represent a highly heterogeneous population that is often analyzed in aggregate, overlooking important clinical differences. By leveraging real-world data, this study enables stratified analysis that captures this complexity, allowing for differentiation by primary tumor site, biomarker status (eg, HPV), and treatment modality (surgical vs nonsurgical). Such detailed characterization supports more tailored clinical insights and advances toward personalized care.

##### Dual Predictive Focus

This study develops 2 complementary predictive models tailored to different stages of HNSCC. The first model aims to identify among patients with localized or locally advanced disease undergoing radical treatment those at higher risk of progression or recurrence who may be candidates for treatment intensification strategies. The second model focuses on patients with recurrent or metastatic disease, seeking to predict response to immunotherapy before initiating first-line systemic treatment, thus informing more personalized therapeutic decisions.

### Limitations

#### Retrospective Design

As an observational retrospective study, there is an inherent risk of bias and confounding factors that cannot be entirely controlled.

#### Reliance on EHR Data Quality

The accuracy of model training and outputs depends on the completeness and consistency of EHR documentation, which may vary across institutions and over time.

#### Data Availability and Standardization

Only information contained in the EHRs can be analyzed. Missing or incomplete data and heterogeneity in EHR structures, terminology, and documentation practices may impact data quality and interpretation across the different databases included in this study, emanating from medical data collection issues. These include the use of standard vs in-house medical terminology, omitted information, or misuse of sections in the records, which are also potential limitations for this protocol to be considered.

### Conclusions

The HNC-TACTIC study implements a layered AI approach in HNC, leveraging cNLP to systematically structure unstructured EHR text into deep-world data encompassing population, treatment, and outcome variables, and subsequently applying ML techniques to this enriched dataset to develop predictive models for treatment response, enabling improved prognostic categorization of patients with HNC. This study will be based on retrospectively extracted data from EHRs from hospitals worldwide, increasing the reliability of the results. By integrating real-world, multilingual clinical data, this study may contribute to a better understanding of disease trajectories in HNSCC and support the future development of prognostic tools that could inform clinical research and, ultimately, patient management.

## Supplementary material

10.2196/83598 Multimedia Appendix 1 Table S4 Sample size estimations for Primary Objective 2

## Abbreviations

AI: artificial intelligence
cNLP: clinical natural language processing
EHR: electronic health record
HNC: head and neck cancer
HNC-TACTIC: Toward a Better Paradigm for Head and Neck Cancer Treatment Applying Artificial Intelligence
HNSCC: head and neck squamous cell carcinoma
HPV: human papillomavirus
*ICD*: *International Classification of Diseases*
ICMJE: International Committee of Medical Journal Editors
LOINC: Logical Observation Identifiers Names and Codes
ML: machine learning
NER-NEL: named entity recognition and named entity linking
OS: overall survival
PD-L1: programmed death-ligand 1
SPIRIT: Standard Protocol Items: Recommendations for Interventional Trials
