# Supplementary material for: Toward a Better Paradigm for Head and Neck Cancer Treatment Applying AI (HNC-TACTIC): Protocol for an International Cohort Study of Electronic Health Records
Source: JMIR Res Protoc. 2026 Jul 13;15:e83598. doi: 10.2196/83598 (PMC13361617; doi:10.2196/83598)
Supplement: Multimedia Appendix 1 [file resprot-v15-e83598-s001.docx]

***Table S4: Sample size estimations for Primary Objective 2***

|  | **Number of predictor parameters** | | |
| --- | --- | --- | --- |
| **AUC** | **20** | **30** | **40** |
| **0.65** | 3,926 | 5,889 | 7,852 |
| **0.75** | 1,332 | 1,998 | 2,664 |
| **0.85** | 617 | 925 | 1,233 |

*AUC: Area under the curve*

*Given these values and keeping the current assumptions that 40% of the study cohort will present metastatic disease at some point during the study period, of which 20% will be treated with immunotherapy, the minimum number of recruited patients with HNSCC required to undertake primary objective 2, in a model with an AUC of 0.75 trained with 30 predictor parameters, would be approximately 24,975.*
